# Vulval Intraepithelial Neoplasia: A 15-Year Review of Treatment Outcomes in a UK Centre

**DOI:** 10.7759/cureus.95074

**Published:** 2025-10-21

**Authors:** Mark A McGowan, Krishnayan Haldar, Pubudu Pathiraja, Jane Sterling, Peter Baldwin

**Affiliations:** 1 Obstetrics and Gynaecology, C W Wiebe Medical Centre, Winkler, CAN; 2 Gynaecological Oncology, Cambridge University Hospitals NHS Foundation Trust, Cambridge, GBR; 3 Dermatology, Cambridge University Hospitals NHS Foundation Trust, Cambridge, GBR

**Keywords:** immunomodulator, precancerous condition, surgical skin excision, vulval lesion, vulvar intraepithelial neoplasia

## Abstract

Background

Vulvar intraepithelial neoplasia (VIN) is a premalignant condition with a high risk of recurrence. Understanding recurrence patterns may help guide treatment planning and follow-up, particularly when comparing unifocal and multifocal disease.

Methods

We conducted a 15-year retrospective cohort study of patients treated for VIN at Cambridge University Hospitals, a tertiary referral center in Cambridge, England, from 2008 to 2022. Patients received local excision (clear or positive margins), CO₂ laser ablation, or medical therapy (imiquimod/cidofovir) and were stratified into unifocal or multifocal subgroups. Demographic and clinical characteristics were analyzed, with recurrence rates and time to recurrence compared across groups.

Results

A total of 108 patients were included: 26 treated with local excision with clear margins, 44 with excision with positive margins, 25 with laser, and 13 with medical therapy. The median age was 51 years (95% CI 50-55). In unifocal disease, time to recurrence differed significantly by treatment modality (p = 0.04), with the longest median interval in patients with clear-margin excision. In multifocal disease, recurrence risk remained high across all treatment groups without significant differences in time to recurrence (p = 0.21).

Conclusions

Unifocal VIN treated with excision and negative margins was associated with the lowest recurrence risk, supporting the possibility of reduced follow-up intensity in selected cases. By contrast, positive margins and multifocal disease were linked with earlier and more frequent recurrence, underscoring the importance of close, long-term surveillance. These findings may inform follow-up strategies, although further research is needed.

## Introduction

Vulval intraepithelial neoplasia (VIN) is a premalignant lesion that occurs in the vulval skin. In contrast to cervical premalignant lesions, most VIN is high grade, i.e., VIN2 and VIN3 [1]. VIN patients are usually symptomatic with pruritus, pain, and dyspareunia. The incidence of VIN is increasing and is estimated to be 3.8 per 100,000 women-years [2]. Differentiated VIN (dVIN) is more common in older women and is HPV-independent, arising in association with lichen sclerosus or, less commonly, lichen planus [3]. The increasing prevalence of high-risk HPV, associated with undifferentiated VIN, explains the recent rise in VIN cases [4]. While HPV vaccination programmes in developed countries are expected to reduce the incidence of undifferentiated VIN, it is probable to take decades to achieve their full impact [5].

The concern regarding potential malignant transformation of VIN favours active treatment of high-grade squamous intraepithelial lesions and dVIN rather than observation. The risk of progression of VIN varies between studies and the type of VIN but is likely to be 5-25% for HPV-associated diseases [2,6]. Therapeutic interventions may be medical or surgical. Topical immunotherapy with imiquimod or cidofovir is effective, but cidofovir may achieve a better long-term response [7]. Surgical options for treatment include local excision (LE) or laser ablation, typically with a CO2 laser.

Data regarding recurrence risk after surgical treatments are conflicting. A large retrospective study reported recurrence rates of 24% in the laser ablation group [8]. Even with negative margins, the recurrence rate of VIN within one year after primary LE can reach as high as 50%, according to the literature [9]. Experience with photodynamic therapy to treat VIN is limited and mainly relates to multifocal disease (MFD) [10]. Studies suggest that treatment with immunotherapy, such as cidofovir or imiquimod, has shown promising short-term efficacy, although longer-term data remain limited [11]. A recent systematic review concluded that the mode of treatment, either medical or surgical, does not significantly impact the risk of recurrence [12].

Following primary treatment, i.e., medical or surgical by excision, complete initial response rates are high, from 20% to 40% [13]. The initial response rate is defined as the complete clinical and histological clearance of VIN at the initial follow-up. Factors associated with recurrence are MFD [9], persistent HPV-positive status [14], concomitant lichen sclerosus [2], and positive surgical margins [15].

A review of international guidelines reveals a lack of consensus on the most appropriate follow-up regimen. For UFD (unifocal disease) and MFD, the British Gynaecological Cancer Society (BGCS) suggests follow-up at six-monthly intervals in the first two years and yearly thereafter for a total of five years [5]. At five years, UFD with no recurrence could be considered for discharge, but for MFD, long-term follow-up is suggested. Consensus statements on pre-invasive vulval disease from international societies which include the European Society of Gynaecological Oncology (ESGO), International Society for Vulvovaginal Disease (ISSVD), European College for Study of Vulval Disease (ECSVD), and European Federation for Colposcopy (EFC) suggest decisions on follow-up should be based primarily on the recurrence risk whilst also emphasising the importance of patient education [16]. The American College of Obstetrics and Gynaecology (ACOG) recommends review at six and 12 months in VIN patients following treatment and annual visual inspection thereafter [17]. Whilst follow-up schedules vary between countries, the current consensus is to offer long-term follow-up of VIN, with the aim of preventing disease progression by detecting recurrences early.

Outpatient consultations in the UK National Health Service (NHS) represent 5.5% of the annual expenditure (£8.6 billion) [18]. There has been increasing pressure on the UK healthcare system in relation to clinic capacity, particularly following the COVID-19 pandemic [19,20]. For VIN, the optimum follow-up regime should focus resources on patients at high risk of recurrence and at the time when recurrence is most likely. This study aims to optimise the use of outpatient resources in publicly funded healthcare systems by analysing recurrence patterns following medical and surgical treatments for vulvar intraepithelial neoplasia (VIN), with the goal of informing evidence-based and sustainable follow-up strategies.

This article was previously presented as a meeting abstract at the 2023 European Society of Gynaecological Oncology Annual Scientific Meeting on September 28, 2023.

## Materials and methods

We conducted a 15-year retrospective cohort study of medically and surgically treated VIN cases at a tertiary referral centre, Cambridge University Hospitals, from 11th of July 2008 to 7th of October 2022. All cases were managed by UK subspecialty-accredited gynaecological oncologists and/or dermatologists with a special interest in vulval disease. Our centre has a multidisciplinary specialist vulval clinic for managing complex vulval patients.

Cases were identified by a search of our electronic pathology database (N = 265) and our specialist vulval clinic database (N = 54). Cases were evaluated using patient letters, surgical documentation, and pathology reports from the integrated electronic health record system to evaluate the timing of first VIN (histologically confirmed), treatment modalities, and timing of progression to cancer. The CO2 laser was exclusively employed for laser treatments at our institution.

**Figure 1:**
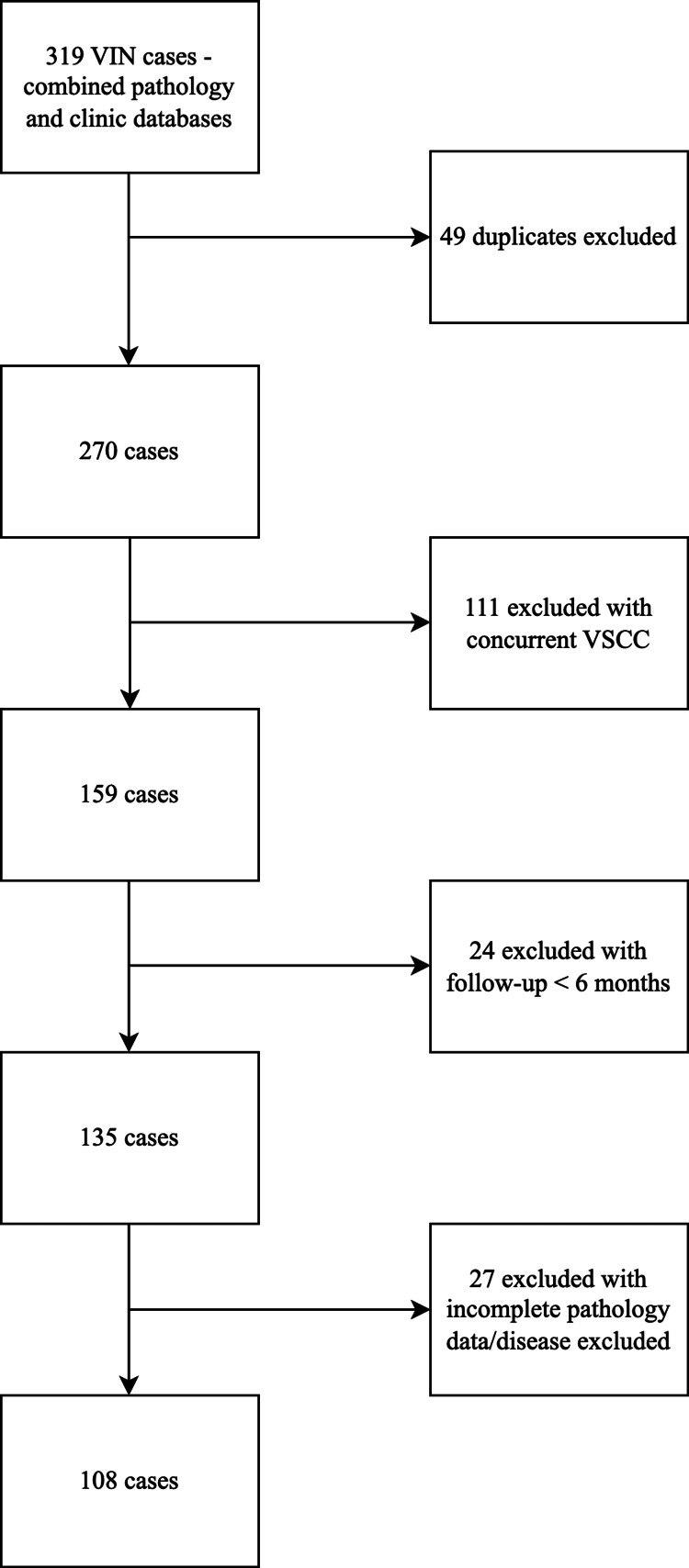
Study flow diagram

A total of 319 cases were identified for evaluation, with 108 cases for analysis after exclusions. Exclusion criteria included duplicates removed upon combining clinic and pathology databases (N = 49), concomitant vulval cancer identified within the VIN pathology specimen (N = 111), follow-up <6 months (N = 24), incomplete pathology information, or anogenital disease but no VIN in the specimen (N = 27). We excluded cases with less than six months of follow-up because recurrences are uncommon for both UFD and MFD VIN groups during this initial period. Analysis of excluded cases revealed no recurrences within the short follow-up period of less than six months. A total of 211 cases were excluded from the analysis (see Figure 1).

Cases were followed every six months for the first two years, then yearly thereafter for the next three years. Immunosuppression was defined as any patient on immunosuppressive drug therapy, e.g., for autoimmune disease, following organ transplant, or any patient with human immunodeficiency virus (HIV) at diagnosis.

Categorical variables were compared using a Chi-squared analysis. Continuous variables were compared using a Kruskal-Wallis test. A Chi-squared and Cox proportional hazard analysis were used to analyse risk factors with a significance level of 0.05. All statistical tests were two-sided. The primary outcome of recurrence across treatment modalities was compared using the Kaplan-Meier method to establish a follow-up schedule. Univariate associations between variables and survival were analysed using the log-rank test. All statistical calculations were performed using IBM SPSS Statistics for Windows, version 26.0 (IBM Corp., Armonk, NY).

All procedures performed in this study involving human participants were in accordance with the ethical standards of the institutional research committee and with the 1964 Helsinki declaration and its later amendments or comparable ethical standards. Given its retrospective nature and anonymised patient data, the need for informed consent was waived.

## Results

The study included 108 cases of high-grade VIN. The median age of the cohort was 51 years (95% CI 50-55). According to primary treatment, 26 (24%) cases were in group 1 (LE with clear margins, LE-), 44 (41%) in group 2 (LE with positive margins, LE+), 25 (23%) in group 3 (CO₂ laser), and 13 (12%) in group 4 (medical therapy; 10 imiquimod and 3 cidofovir). Patient characteristics and histology are summarized in Table 1.

**Table 1:** Patient characteristics and histology H: Kruskal-Wallis test for continuous variables, χ²: Chi-square test for categorical variables *: For VIN histological subtypes, a single χ² test was applied to the overall distribution across treatment groups. The result is shown once under the dVIN row; other rows are presented for completeness without separate test statistics. p < 0.05 is considered statistically significant.

Parameter	Group 1 (LE–) (n = 26, 24%)	Group 2 (LE+) (n = 44, 41%)	Group 3 (Laser) (n = 25, 23%)	Group 4 (Medical) (n = 13, 12%)	Test statistic	p-value
Median age (years, 95% CI)	57 (52–62)	52 (50–58)	47 (44–52)	48 (47–56)	H(3) = 9.3	0.03
Histology						
dVIN	0 (0%)	1 (2%)	0 (0%)	1 (2%)	χ²(6, N = 108)	0.55
VIN1	0 (0%)	0 (0%)	0 (0%)	0.0 (0%)	*	-
VIN2	1 (4%)	5 (11%)	5 (20%)	2 (15%)	*	-
VIN3	25 (96%)	38 (86%)	20 (80%)	11 (85%)	*	-
Median follow-up (months, 95% CI)	46 (32–60)	42 (36–51)	53 (42–64)	40 (29–65)	H(3)=2.7	0.4
Smoking	7 (27%)	11 (27%)	7 (41%)	3.0 (23%)	χ²(3, N = 108) = 3.0	0.4
Immunosuppression	2 (8%)	5 (11%)	4 (24%)	0 (0%)	χ²(3, N = 108) = 4.64	0.2
Lichen sclerosus	3 (12%)	5 (11%)	1 (6%)	1 (8%)	χ²(3, N = 108)=1.8	0.6
AIN	2 (8%)	2 (5%)	3 (12%)	2 (15%)	χ²(3, N = 108)=3.0	0.4
CIN	2 (8%)	6 (14%)	1 (4%)	0 (0%)	χ²(3, N = 108)=2.4	0.5
VaIN	1 (4%)	3 (7%)	2 (8%)	0 (0%)	χ²(3, N = 108)=0.4	0.9
Multicentric disease (≥2 sites)	1 (4%)	3 (7%)	1 (4%)	2 (15%)	χ²(3, N = 108)=2.4	0.5

The median follow-up period did not differ significantly between treatment groups: 46 months (95% CI 32-60) in group 1, 42 (36-51) in group 2, 53 (42-64) in group 3, and 40 (29-65) in group 4 (H(3) = 2.7, p = 0.4; Table 1).

Univariable Cox proportional-hazards analysis was performed to explore the association between potential confounders and time to recurrence (Table 2). None of the examined factors, including smoking (HR 1.9, 95 % CI 0.5-7.5, p = 0.36), immunosuppression (HR 2.1, 95 % CI 0.5-8.2, p = 0.30), lichen sclerosus (HR 1.4, 95 % CI 0.35-5.52, p = 0.63), concomitant HPV (HR 1.5, 95 % CI 0.39-6.00, p = 0.55), CIN (HR 1.8, 95 % CI 0.46-7.17, p = 0.39), VaIN (HR 1.2, 95 % CI 0.25-5.96, p = 0.78), or multicentric VIN (HR 1.3, 95 % CI 0.26-6.38, p = 0.73), showed a statistically significant association with recurrence. On Cox regression, no baseline clinical or pathological covariates demonstrated a significant effect on recurrence, supporting that the observed differences between treatment groups on Kaplan-Meier analysis were not driven by confounding factors.

**Table 2:** Univariable Cox proportional-hazards regression for time to recurrence Univariable Cox proportional-hazards models were fitted for each covariate using time-to-recurrence as the outcome. HR: hazard ratio, CI: confidence interval. p-values from the Wald test. A multivariable Cox model was not performed due to the limited number of recurrence events, which would risk model overfitting and instability. p < 0.05 is considered statistically significant.

Variable	Hazard ratio (95% CI)	p-value
Smoking	2.0 (0.5–7.5)	0.4
Immunosuppression	2.1 (0.5–8.2)	0.3
Lichen sclerosus	1.4 (0.4–5.5)	0.6
AIN	1.28 (0.26–6.38)	0.7
CIN	1.8 (0.5–7.2)	0.4
VaIN	1.2 (0.3–6.0)	0.8
Multicentric disease (≥2 sites)	1.3 (0.3–6.4)	0.7

A significant difference in disease distribution was observed between treatment groups, with unifocal disease more common in group 1 and multifocal disease more frequent in groups 2 and 3 (χ²(3, N = 108) = 11.3, p = 0.01; Table 3). Recurrence rates did not differ significantly between treatment groups for either unifocal disease (χ²(3, N = 60) = 6.3, p = 0.1; Table 3) or multifocal disease (χ²(3, N = 49) = 4.6, p = 0.2; Table 3).

**Table 3:** Unifocal and multifocal analysis H: Kruskal–Wallis test for continuous variables (time to recurrence), χ²: Chi-square test for categorical variables. *: Unifocal versus multifocal disease distribution across treatment groups was evaluated using a single χ² test. The result is reported once under the unifocal row; the multifocal row is included for completeness without a separate test statistic. p < 0.05 is considered statistically significant.

Parameter	Group 1 (LE–)	Group 2 (LE+)	Group 3 (Laser)	Group 4 (Medical)	Test statistic	p-value
No. of unifocal cases (% of group)	21 (81%)	22 (50%)	9 (35%)	7 (54%)	χ²(3, N = 108)=11.3	0.01
No. of multifocal cases (% of group)	5 (19%)	22 (50%)	16 (64%)	6 (46%)	*	-
No. of unifocal 1st recurrences (% of group)	4 (19%)	11 (50%)	5 (56%)	2 (29%)	χ²(3, N = 60)=6.3	0.1
No. of multifocal 1st recurrences (% of group)	1 (20%)	13 (59%)	12 (75%)	5 (83%)	χ²(3, N = 49) = 4.6	0.2
Median time to 1st unifocal recurrence (months, 95% CI)	68 (CI not estimable)	23 (10–33)	11 (CI not estimable)	17 (CI not estimable)	H(3)=8.1	0.04
Median time to 1st multifocal recurrence (months, 95% CI)	34 (CI not estimable)	22 (16-28)	26 (17-38)	22 (10-29)	H(3)=4.6	0.2

**Figure 2:**
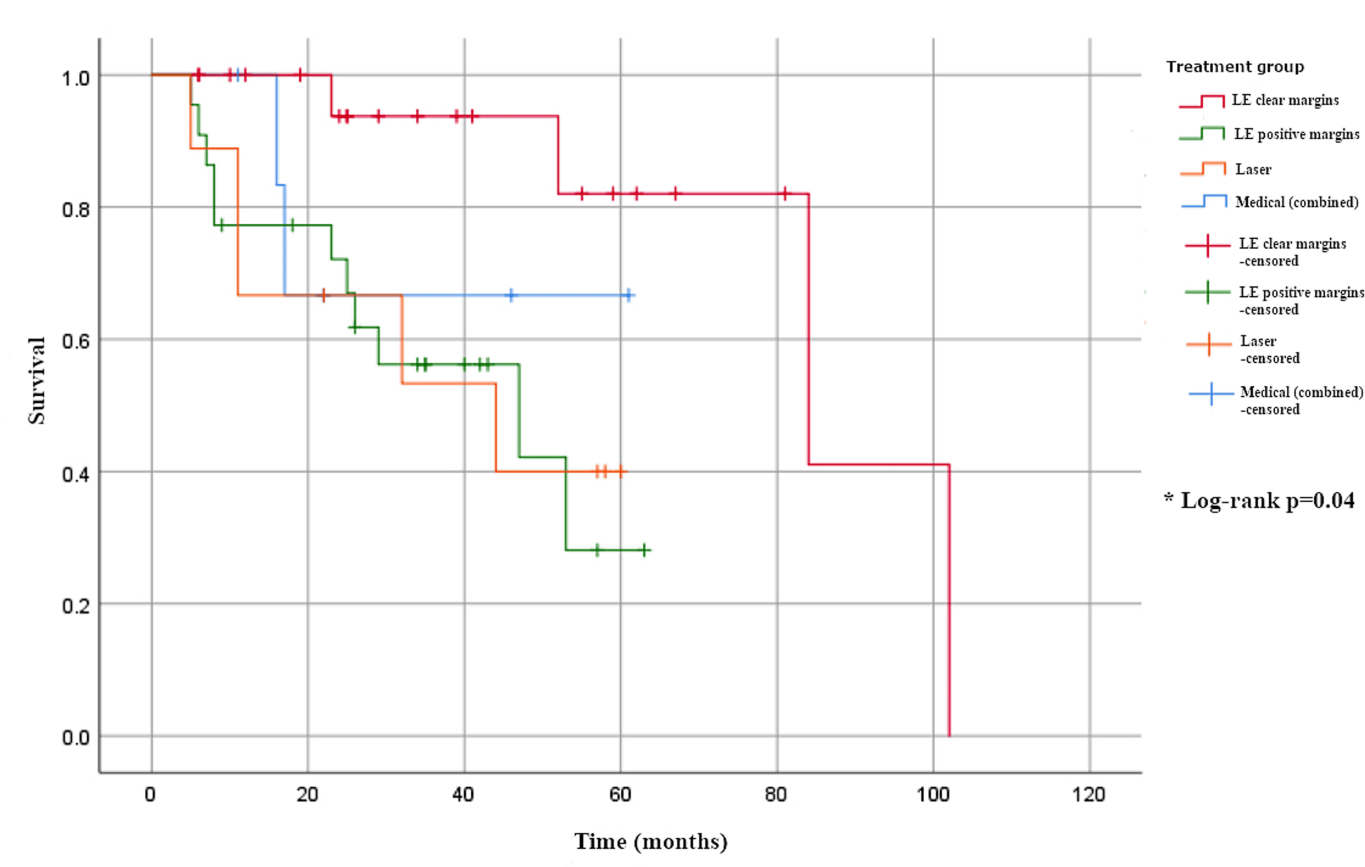
Time to recurrence in unifocal disease.

**Figure 3:**
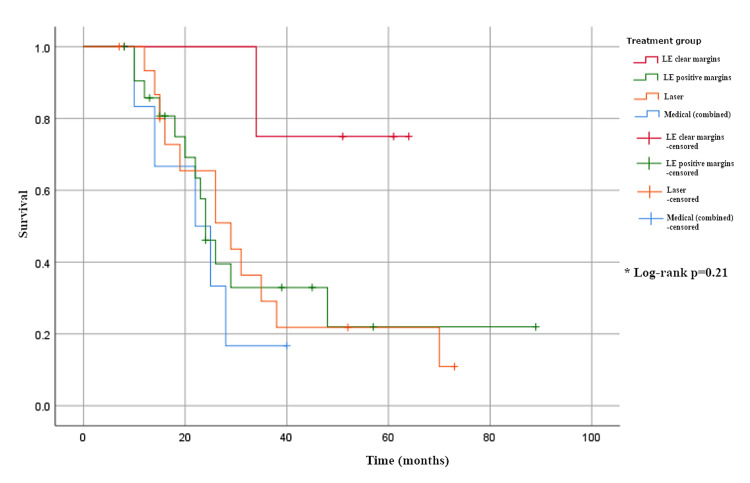
Time to recurrence in multifocal disease.

Time to first recurrence varied significantly in unifocal disease (H(3) = 8.1, p = 0.04), with the longest median interval observed in group 1 (68 months, CI not estimable) compared to 23 months (95% CI 10-33) in group 2, 11 months (CI not estimable) in group 3, and 17 months (CI not estimable) in group 4 (Table 3, Figure 2). By contrast, time to recurrence in multifocal disease showed no significant difference across treatment groups (H(3) = 4.6, p = 0.2), with median times of 34 months (CI not estimable) in group 1, 22 months (16-28) in group 2, 26 months (17-38) in group 3, and 22 months (10-29) in group 4 (Table 3, Figure 3).

## Discussion

From this 15-year study, we can be reassured by the outcomes following LE of UFD, where clear margins are achieved: group 1, LE-. This group shows a low recurrence rate and a long interval to recurrence. It is likely that the “recurrences” represent a new disease, given the long median time to recurrence of 68 months (5.7 years). It should be noted that this new disease/recurrence interval falls outside the current UK BGCS five-year follow-up schedule. Patient education and easy access to re-referral are important if presentation with a "new" disease is not to be delayed.

**Figure 4:**
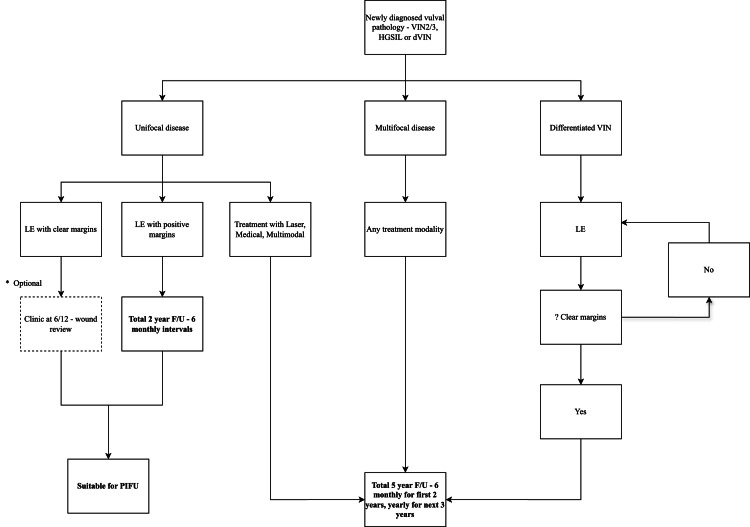
Suggested follow-up schedule for premalignant vulval pathology based on study data

Analysis of our UFD excised with positive margins (LE+) showed these recurrences occurred early on (Figure 2). This provides a potential opportunity for reducing long-term follow-up for patients with UFD, even where margins are positive. Figure 4 shows a customised follow-up plan aligned with the findings of this research to enhance healthcare economics. Upon completing the follow-up schedule, we would advocate additional patient education with a view to discharge to patient-initiated follow-up (PIFU) [21].

The data presented support the view that MFD should be viewed as a chronic condition with high recurrence rates of 49/108 (45%) from our data. The choice of first treatment modality did not appear to influence the number of recurrences (p = 0.21 for median time to first recurrence). When surgical excision is employed, involved microscopic margins are common, i.e., 44/70 (63%) in combined UFD/MFD groups. Similarly, recurrence rates were high in the excisional group with positive margins, with the majority occurring within the first two years of 11/22 (50%) for UFD and 13/22 (59%) for MFD. Repeat recurrences were also relatively common in this group. We suggest that the choice of treatment should be predicated on symptom control and maintaining function. The surgical approach of "excision for clear margins" appears flawed for MFD due to the high risk of recurrence. Quality of life studies for patients undergoing vulval excision are limited, but the procedure is associated with sexual dysfunction and anatomical loss, both of which are important considerations in younger patients [22]. We advocate individualised treatment, including the use of phased and/or multimodal therapy, to reduce long-term treatment-related morbidity in the management of MFD.

Previous studies have suggested a correlation between LE with positive margins (group 2 - LE+) and higher rates of VIN recurrence compared with LE where clear margins are obtained (group 1 - LE-), with authors recommending close follow-up of this cohort [15,23,24]. Our study supports the higher risk of recurrence with involved surgical margins, particularly for MFD - group 2 - LE+; 59%). We would agree that where clear margins are not achieved, recurrence risks are high and follow-up is required.

Two large retrospective studies demonstrate progression rates following VIN treatment of 2.2-6.7% [23,25], which align with the findings of this study. The long duration of this study's follow-up period allows for the evaluation of the progression to cancer. Our study provides further reassurance about the low risk of progression to cancer following LE with clear margins, i.e., Group 1- LE-, which showed no progression in either unifocal or multifocal subgroups. The LE with positive margins (group 2 - LE+) had a progression rate of 7%, which is consistent with that reported in the literature. Group 3 (laser) had progression rates of 8%, also consistent with the published literature of 7% [26]. However, no firm conclusions can be drawn regarding the primary treatment medical group due to the small number of patients (group 4). The literature advises caution in non-responders to immunotherapy because of the risk of progression [27]. We would support the recommendation for local excision for this group of non-responders.

In this study, dVIN is underrepresented in the patient cohort, with just one case, but accounting for 5% of the excluded cases. The literature shows dVIN has a higher malignant potential, more frequently found on pathological review of vulvar cancer specimens [28]. We therefore recommend LE with clear margins for any dVIN histology and a follow-up schedule as per Figure 4.

Small centralised specialist UK vulval clinics take regional referrals and have high demands with limited capacity. Even small reductions in follow-up requirements may have a significant impact on these services. Also, the NHS Long Term Plan has the aim of reducing the number of face-to-face outpatient follow-ups to reduce healthcare expenditure. In the NHS, the cost of an outpatient colposcopy appointment is £238 per patient [18]. Based on our results, we have suggested a possible treatment algorithm (Figure 4).

Our data support PIFU for local excision of unifocal lesions with clear margins (group 1, LE-), which comprised 21 patients (19% of the total cohort). These patients have low recurrence risks and may be discharged earlier. There are potential cost savings of £1428 per patient and £30k when applied to this entire cohort over five years, with just one follow-up at six months compared to the current BGCS schedule of six-monthly follow-up for two years and then yearly follow-up for the next three years. 

Assessing the group with UFD and positive surgical margins (group 2, LE+), recurrences are typically early (within two years), and limiting the length of follow-up could be considered. In the current study, this strategy would affect 22 patients (20% of the total cohort) with a potential saving of £714 per patient and £15k when applied to the cohort over a five-year period.

By implementing our recommended follow-up schedule, the total cost savings would be £45k when applied to the whole cohort. This substantial financial benefit suggests that a national rollout would yield significant healthcare savings.

Within the UK, there has been increasing pressure on gynaecological oncology clinics following the pandemic. Whilst the study aims to reduce the cost of follow-up following VIN treatment, the oncological safety of recurrences in PIFU patients is an important consideration. From the analysis of our data, we have limited PIFU to patients treated for unifocal disease. In the LE - margin group of patients, we had 4/22 (19%) recurrences over the 15-year follow-up period.

In the UK, VIN is typically managed by subspecialty-trained gynecological oncologists or gynecologists with expertise in managing early-stage cancers. After a diagnosis of VIN, consultations are often supplemented with patient literature outlining the condition and providing information on symptoms of recurrence following treatment. A recent study also emphasises the importance of patient education on normal vulvar anatomy as an integral part of consultations on vulval diseases [29]. In the UK, cancer nurse specialists (CNS) also play a crucial role in supporting patients through electronic communication, face-to-face consultations, and helping them gain confidence in self-examination. They also serve as a key point of contact for concerns regarding vulval disease recurrence.

For treated UFD patients, careful selection for PIFU is crucial. PIFU should prioritise those who are confident in their understanding of vulvar anatomy, capable of recognising symptoms of recurrence, and adept at performing self-examinations. For UFD patients with significant recurrence risk factors such as immunosuppression, we recommend consultant-led follow-up rather than PIFU. Similarly, for patients with cognitive impairment, there is a risk of delayed disease presentation, and consultant-led follow-up is preferable to PIFU. Patients eligible for the PIFU pathway should be offered the choice between consultant-led follow-up and PIFU, ensuring their preferences are considered. For patients on the PIFU pathway, we advise an expedited pathway for clinic review in the event of recurrence symptoms, facilitated by the CNS team.

The main weakness of this study is its retrospective data and the heterogeneity of follow-up data. VIN recurrences may have occurred earlier than the time of clinical detection or presentation. Although the intended follow-up schedule (six-monthly for two years, then annually) was standardised, adherence varied among patients and was influenced by both patient availability and clinician scheduling. Differences in access to care and appointment intervals may therefore have led to variable timing in recurrence detection, introducing potential detection bias.

Interpreting outcomes for the primary treatment modalities should be made with some caution; these may be influenced by both the amount and location of disease. Thus, well-lateralised, small unifocal lesions will naturally tend to be excisional candidates. Similarly, UFD at challenging anatomical locations, e.g., peri-clitoral, are often preferentially treated with laser ablation/medical therapy. This may introduce bias to the recurrence risk data, with surgical excision being less likely to be employed for more challenging disease locations. Lastly, although our study is limited by a relatively small cohort size due to the rarity of VIN (including dVIN), it provides valuable insights into recurrence timing and follow-up economics in the absence of larger, adequately powered studies on this topic. The medical treatment groups are particularly underpowered owing to their small sample sizes. The small numbers reflect the rarity of VIN and dVIN presentations in a regional referral population over a 15-year period.

This was a single-centre study conducted in a tertiary UK referral centre, and findings may not be generalisable to other settings with different care pathways, patient demographics, or healthcare resource structures. Institutional costings are also likely to vary between centres and healthcare systems, and the financial estimates presented here should be interpreted accordingly. Larger, multicentre or prospective population-based studies are warranted to validate these findings and confirm the optimal follow-up intervals.

A key strength of our study lies in the combination of the 15-year study period and the regular follow-up of our treatment cases. This study thus allows for a more reliable evaluation of recurrence rates in this rare disease. Also, at our institution, we use an electronic medical record, which improves data integrity through scrupulous analysis of pathology reports, operative records, and clinic letters/notes.

## Conclusions

Outcomes in VIN differ according to both treatment modality and disease distribution. Local excision with negative margins was associated with a low recurrence risk, supporting earlier discharge and PIFU in carefully selected patients. In contrast, positive margins were linked to early recurrence, most often within the first two years, underscoring the importance of closer surveillance during this interval. Multifocal disease remains a particular challenge, with high recurrence rates and shorter recurrence times regardless of treatment, highlighting the need for prolonged and individualized follow-up.

These findings emphasize the value of tailoring follow-up to disease pattern and margin status, balancing patient safety with more efficient use of specialist services. Future research should aim to refine management strategies for multifocal disease and incorporate prospective data to validate tailored follow-up pathways, including PIFU models that assess adherence to self-examination and safety outcomes.

## References

[1] Maniar KP, Ronnett BM, Vang R, Yemelyanova A (2013). Coexisting high-grade vulvar intraepithelial neoplasia (VIN) and condyloma acuminatum: independent lesions due to different HPV types occurring in immunocompromised patients. Am J Surg Pathol.

[2] Thuijs NB, van Beurden M, Bruggink AH, Steenbergen RD, Berkhof J, Bleeker MC (2021). Vulvar intraepithelial neoplasia: incidence and long-term risk of vulvar squamous cell carcinoma. Int J Cancer.

[3] Lebreton M, Carton I, Brousse S, Lavoué V, Body G, Levêque J, Nyangoh-Timoh K (2020). Vulvar intraepithelial neoplasia: classification, epidemiology, diagnosis, and management. J Gynecol Obstet Hum Reprod.

[4] Bray F, Laversanne M, Weiderpass E, Arbyn M (2020). Geographic and temporal variations in the incidence of vulvar and vaginal cancers. Int J Cancer.

[5] Morrison J, Baldwin P, Buckley L (2020). British Gynaecological Cancer Society (BGCS) vulval cancer guidelines: recommendations for practice. Eur J Obstet Gynecol Reprod Biol.

[6] van de Nieuwenhof HP, Massuger LF, van der Avoort IA (2009). Vulvar squamous cell carcinoma development after diagnosis of VIN increases with age. Eur J Cancer.

[7] Hurt CN, Jones S, Madden TA (2018). Recurrence of vulval intraepithelial neoplasia following treatment with cidofovir or imiquimod: results from a multicentre, randomised, phase II trial (RT3VIN). BJOG.

[8] Fehr MK, Baumann M, Mueller M, Fink D, Heinzl S, Imesch P, Dedes K (2013). Disease progression and recurrence in women treated for vulvovaginal intraepithelial neoplasia. J Gynecol Oncol.

[9] van Esch EM, Dam MC, Osse ME (2013). Clinical characteristics associated with development of recurrence and progression in usual-type vulvar intraepithelial neoplasia. Int J Gynecol Cancer.

[10] Tosti G, Iacobone AD, Preti EP, Vaccari S, Barisani A, Pennacchioli E, Cantisani C (2018). The role of photodynamic therapy in the treatment of vulvar intraepithelial neoplasia. Biomedicines.

[11] Pepas L, Kaushik S, Nordin A, Bryant A, Lawrie TA (2015). Medical interventions for high-grade vulval intraepithelial neoplasia. Cochrane Database Syst Rev.

[12] Jamieson A, Tse SS, Brar H, Sadownik LA, Proctor L (2022). A systematic review of risk factors for development, recurrence, and progression of vulvar intraepithelial neoplasia. J Low Genit Tract Dis.

[13] Hillemanns P, Wang X, Staehle S, Michels W, Dannecker C (2006). Evaluation of different treatment modalities for vulvar intraepithelial neoplasia (VIN): CO(2) laser vaporization, photodynamic therapy, excision and vulvectomy. Gynecol Oncol.

[14] Nitecki R, Feltmate CM (2018). Human papillomavirus and nonhuman papillomavirus pathways to vulvar squamous cell carcinoma: a review. Curr Probl Cancer.

[15] Modesitt SC, Waters AB, Walton L (1998). Vulvar intraepithelial neoplasia III: occult cancer and the impact of margin status on recurrence. Obstet Gynecol.

[16] Preti M, Joura E, Vieira-Baptista P (2022). The European Society of Gynaecological Oncology (ESGO), the International Society for the Study of Vulvovaginal Disease (ISSVD), the European College for the Study of Vulval Disease (ECSVD) and the European Federation for Colposcopy (EFC) consensus statements on pre-invasive vulvar lesions. Int J Gynecol Cancer.

[17] (2016). Committee Opinion No. 675 Summary: management of vulvar intraepithelial neoplasia. Obstet Gynecol.

[18] (2023). NHS England - National schedule of NHS costs. https://www.england.nhs.uk/costing-in-the-nhs/national-cost-collection/.

[19] (2023). NHS: the NHS long term plan. https://www.longtermplan.nhs.uk/publication/nhs-long-term-plan/.

[20] Levell NJ (2022). NHS outpatient secondary care: a time of challenges and opportunities. Future Healthc J.

[21] Newton C, Nordin A, Rolland P (2020). British Gynaecological Cancer Society recommendations and guidance on patient-initiated follow-up (PIFU). Int J Gynecol Cancer.

[22] Aerts L, Enzlin P, Vergote I, Verhaeghe J, Poppe W, Amant F (2012). Sexual, psychological, and relational functioning in women after surgical treatment for vulvar malignancy: a literature review. J Sex Med.

[23] Jones RW, Rowan DM, Stewart AW (2005). Vulvar intraepithelial neoplasia: aspects of the natural history and outcome in 405 women. Obstet Gynecol.

[24] Andreasson B, Bock JE (1985). Intraepithelial neoplasia in the vulvar region. Gynecol Oncol.

[25] Satmary W, Holschneider CH, Brunette LL, Natarajan S (2018). Vulvar intraepithelial neoplasia: risk factors for recurrence. Gynecol Oncol.

[26] Herod JJ, Shafi MI, Rollason TP, Jordan JA, Luesley DM (1996). Vulvar intraepithelial neoplasia: long term follow up of treated and untreated women. Br J Obstet Gynaecol.

[27] Fernández-Montolí ME, Heydari F, Lavecchia F (2022). Vulvar high-grade squamous intraepithelial lesions treated with imiquimod: can persistence of human papillomavirus predict recurrence?. Cancers (Basel).

[28] Singh N, Leen SL, Han G (2015). Expanding the morphologic spectrum of differentiated VIN (dVIN) through detailed mapping of cases with p53 loss. Am J Surg Pathol.

[29] Preti M, Selk A, Stockdale C (2021). Knowledge of vulvar anatomy and self-examination in a sample of Italian women. J Low Genit Tract Dis.

